# Maternal stress during pregnancy alters fetal cortico-cerebellar connectivity in utero and increases child sleep problems after birth

**DOI:** 10.1038/s41598-021-81681-y

**Published:** 2021-01-26

**Authors:** Marion I. van den Heuvel, Jasmine L. Hect, Benjamin L. Smarr, Tamara Qawasmeh, Lance J. Kriegsfeld, Jeanne Barcelona, Kowsar E. Hijazi, Moriah E. Thomason

**Affiliations:** 1 Department of Cognitive Neuropsychology, Tilburg University, Tilburg, The Netherlands; 2 Department of Psychology, Wayne State University, Detroit, MI USA; 3 Department of Bioengineering and Halicioglu Data Science Institute, UCSD, San Diego, CA USA; 4 Department of Psychology, University of California Berkeley, Berkeley, CA USA; 5 Helen Wills Neuroscience Institute, University of California Berkeley, Berkeley, CA USA; 6 Department of Kinesiology, Health, and Sport Studies, Wayne State University, Detroit, MI USA; 7 Department of Child and Adolescent Psychiatry, New York University Grossman School of Medicine, NYU Langone Medical Center, New York, USA; 8 Department of Population Health, New York University Grossman School of Medicine, NYU Langone Medical Center, New York, USA

**Keywords:** Stress and resilience, Sleep disorders

## Abstract

Child sleep disorders are increasingly prevalent and understanding early predictors of sleep problems, starting in utero, may meaningfully guide future prevention efforts. Here, we investigated whether prenatal exposure to maternal psychological stress is associated with increased sleep problems in toddlers. We also examined whether fetal brain connectivity has direct or indirect influence on this putative association. Pregnant women underwent fetal resting-state functional connectivity MRI and completed questionnaires on stress, worry, and negative affect. At 3-year follow-up, 64 mothers reported on child sleep problems, and in the subset that have reached 5-year follow-up, actigraphy data (N = 25) has also been obtained. We observe that higher maternal prenatal stress is associated with increased toddler sleep concerns, with actigraphy sleep metrics, and with decreased fetal cerebellar-insular connectivity. Specific mediating effects were not identified for the fetal brain regions examined. The search for underlying mechanisms of the link between maternal prenatal stress and child sleep problems should be continued and extended to other brain areas.

## Introduction

Childhood sleep problems are increasingly prevalent. From 2003 to 2012, inadequate sleep increased from 23 to 36% among 6–9 year old US children^1^. It is reported that 30–40% of children experience problems falling asleep at night^2^ and more than a quarter of all parents of young children underestimate their child’s sleep requirements as they relate to national guidelines^3^. Children from low socio-economic background and minority groups may be especially at risk, given data showing that minority children are more likely to go to bed after 11 pm and sleep fewer hours at night^4^. Additionally, parental knowledge about child sleep is enhanced in higher income, higher education households^3^.

Disturbed sleep in early childhood poses serious consequences for child outcomes and may play an important role in health disparities among minority children^5^. For instance, longitudinal data suggest that sleep disturbance in early childhood is associated with negative adolescent health outcomes, such as increased risk for obesity especially among minority participants of low SES^6^. Neurobiological frameworks also identify sleep as a key factor in emotion processing^7^. In line with this, sleep problems in toddlers have been found to significantly predict internalizing problems at school age^8^. Identifying predictors of sleep problems early in life informs the development of effective early intervention strategies that have the potential to stave off physical and mental health concerns across the lifespan.

The earliest predictors of child sleep disturbance can potentially be found in utero. Given that child hypervigilance and hyper-arousal are associated with prenatal stress exposure^9–12^, it follows that prenatal exposure to maternal stress and negative affect may be a risk factor for the development of early sleep problems. Indeed, a small number of studies have begun to provide evidence for this linkage, reporting sleep problems in children prenatally exposed to maternal mood disturbance^13,14^ and later links to adult insomnia^15^. Interestingly, studies also report altered sleep patterns in fetuses of anxious mothers. For example, Groome et al.^16^ found that fetuses of mothers with high anxiety symptoms spend more time in quiet sleep and exhibited less gross body movements in active sleep. Despite growing evidence of a relationship between the prenatal environment and sleep problems, no study to date has explored neural mechanisms by which maternal stress may affect child sleep patterns *before* birth.

Alterations in fetal brain structure and function are considered one of the key mechanisms of fetal programming by maternal prenatal stress and negative affect^17–20^. Indeed, evidence for altered brain structure and function in relation to maternal stress during pregnancy has accumulated over recent years; for a recent review see^21^. Recent advances in neuroimaging have made it possible to measure properties of fetal functional brain connectivity in utero^22^ and opened possibilities for examining the effect of maternal prenatal stress on fetal brain network development. This advancement in fetal functional brain connectivity also holds implications for predicting long term outcomes in children. Recently, Thomason, et al.^23^ reported the first associations between fetal functional connectivity of the motor system and infant motor development. Here, we study the effects of maternal stress during pregnancy on fetal functional brain connectivity and the mediating role of fetal brain alterations on the association between maternal prenatal stress and postnatal behavioral outcomes (i.e., sleep) in toddlerhood.

Recently, it has been found that the cerebellum plays a key role in the regulation of sleep, in addition to its well-established role in motor functions^24,25^. The cerebellum grows exceptionally fast in the third trimester of pregnancy and is particularly sensitive to prenatal influences at the end of gestation. Our own investigation of the fetal functional brain network identified three highly connected areas, so-called “hubs”, in the cerebellum, emphasizing the importance of this brain area for fetal functional connectivity^26^. Further, the cerebellum is often mentioned as one of the key components of altered functional brain networks found in patients with insomnia and sleep deprivation^27,28^ and research points to a significant role for the cerebellum in sleep-disordered breathing^29,30^. Taken collectively, these findings point to the cerebellum as a critical region of interest that must be considered in the investigation of brain mechanisms in the association between maternal stress during pregnancy and child sleep problems.

Evaluating sleep and identifying sleep problems in early childhood is of critical importance. However, quantifying child sleep behavior can be challenging. Parent’s subjective sleep reports are the most common method for evaluating child sleep^31^, but this approach is not ideal for several reasons. First, subjective sleep reports are only as informative as the individual parent’s knowledge and understanding of their child’s sleep. Although they have been found useful in reporting the behavioral and contextual issues related to sleep onset and disruptions^32^, parent reports lack the capacity measure and track the sleep–wake patterns thought to contribute to physical^33^ and cognitive outcomes^34^ in childhood. Second, subjective measures are not well validated in diverse populations^32^. Given the wide range of limitations in using self-report sleep assessments, objective measures are a desirable alternative. Actigraphy, is one such alternative methodology that takes a multidimensional approach to sleep assessment, captures sleep quantity, quality, architecture and schedule through continuous and extended monitoring. Evidence suggests that actigraphy is best suited for understanding children’s sleep stability and daily rhythms of rest and activity^35^. A growing body of literature has evidenced actigraphy as the golden standard for measuring sleep, especially in children^36,37^. For these reasons, in the present study, we implement actigraphy in a subset of our sample as cross-validation of parental report.

The aim of the current study was to investigate the relationship between maternal stress and negative affect during pregnancy, fetal cortico-cerebellar functional connectivity and toddler’s sleep problems in a predominantly African-American, high-risk sample from Detroit, MI. Our focus on the fetal cerebellum was driven by the increased interest for this brain area by developmental neuroscientists^38^, the fact that the cerebellum grows exceptionally fast in the third trimester of pregnancy and its extreme sensitivity to prenatal influences at the end of gestation^39–42^ and early adversity^43^. Importantly, the cerebellum is also often implicated in sleep disorders^25,27–30^. We first examined whether toddlers prenatally exposed to maternal psychological stress and negative affect experienced increased sleep problems. Sleep problems were reported by the mother for the full group at toddler age and objectively measured using 1-week, 24-h actigraphy measurements in a subsample at preschool age. Next, we investigated how prenatal exposure to maternal stress affected fetal cortico-cerebellar functional connectivity. We also explored sex interactions for the associations between maternal stress and mood during pregnancy, sleep, and fetal functional connectivity, since previous studies have reported sex differences in response to prenatal exposure to maternal stress^44,45^ and differences between males and females in cerebellum development^41^. Finally, we tested whether the association between toddler’s prenatal exposure to maternal stress and sleep problems was based on an indirect effect (mediation) via altered fetal cortico-cereballar functional connectivity. Building on our previous work in the fetal functional brain network^26^, here, we used three fetal hubs in the posterior cerebellum to compute cortico-cerebellar functional connectivity.

## Results

### Prenatal exposure to maternal prenatal NASF is related to child sleep problems

**Figure 1:**
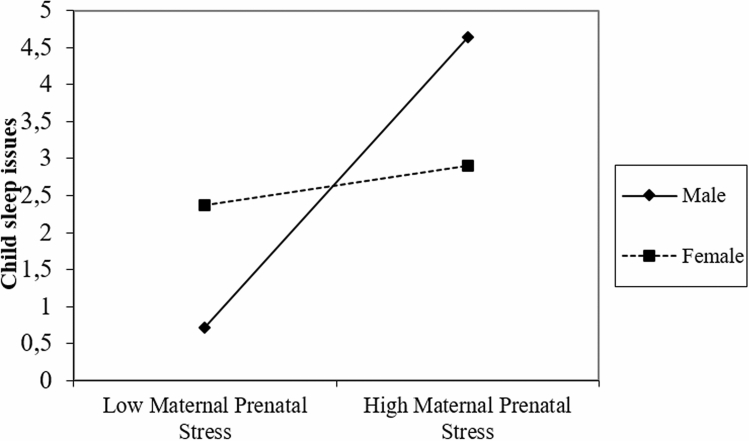
Graphical representation of sex interaction. Sex interaction between maternal prenatal stress and child sleep. Made with excel macro (www.jeremydawson.co.uk/slopes.htm).

Higher maternal prenatal NASF was associated with increased mother-reported sleep problems (*r* = 0.295, *p* = 0.018). Follow-up correlations with individual stress/negative affect scales indicated that the strongest association were found for maternal perceived stress (PSST: *r* = 0.279, *p* = 0.028) and worry (PSWQ: *r* = 0.348, *p* = 0.007) during pregnancy, while no association was found for maternal prenatal depression, life satisfaction and anxiety during pregnancy and child sleep (see “Supplemental Materials” and Table S1 for more details). Additionally, we found a significant interaction with child sex for the association between maternal NASF and child sleep problems (B = − 1.265, *t* = − 2.158, *p* = 0.035). Follow-up testing revealed that the association between maternal NASF during pregnancy and child sleep problems was significant for males only (B = 1.446, *t* = 3.389, *p* = 0.002). Maternal prenatal NASF explained 23.2% of the variance in sleep for males, and only 1% in females. The effect for males remained significant after controlling for gestational age at scan, gestational age and weight at birth, maternal age, and maternal postnatal anxiety at age 3 years. See Fig. 1 for a graphical presentation of the interaction. Regarding the separate stress dimensions, we only observed a similar interactions for perceived stress. We did not observe a sex interaction for maternal satisfaction with life, worry, anxiety, or depression symptoms during pregnancy.

Additionally, we investigated whether objective measures of child sleep were also associated with maternal prenatal NASF in a small follow-up subsample of children who wore an Actigraph wristband for a week at 5 years of age. We found a strong, negative correlation between the maternal prenatal NASF and the ratio between mean circadian and ultradian power (*r* = − 0.408, *p* = 0.043), indicating that objective sleep measures—specifically relative contributions of daily to more frequent behavior structure—also correlated with the maternal prenatal stress factor. However, mean circadian power and mean ultradian power did not yield significant results (*r* = − 0.019, *p* = 0.928; *r* = − 0.178, *p* = 0.395, respectively). Follow-up analyses with separate analyses for different dimensions of maternal stress during pregnancy showed a similar pattern of associations, with a strong association between maternal prenatal depression and the circadian/ultradian power ratio (*r* = − 0.418, *p* = 0.047). Again, no significant association was found between circadian or ultradian power and maternal prenatal depression. To explore why the ratio of circadian/ultradian power is associated with maternal prenatal stress but not the separate measures (circadian and ultradian power), we explored out data further. We found that the standard error of children’s circadian power increased with increasing maternal prenatal stress, especially for maternal prenatal depression (*r* = 0.612, *p* = 0.002). This association seems to indicate that higher maternal stress/depression during pregnancy increased the variability of the children’s circadian power.

### Maternal psychological stress and fetal cortico-cerebellar connectivity

**Figure 2:**
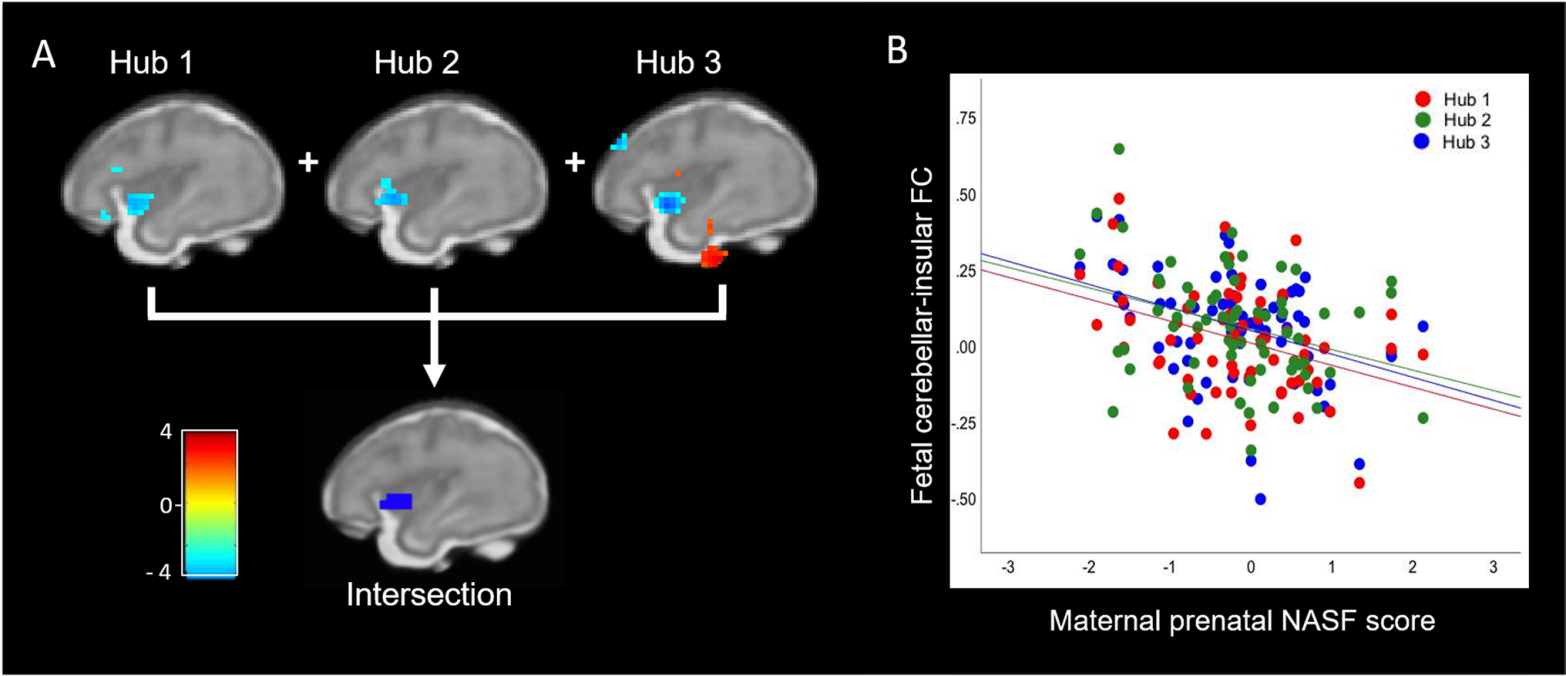
Cerebellar-insular connectivity in relation to maternal prenatal stress and negative affect. Cerebellar-insular connectivity (left insula) was related to maternal psychological stress during pregnancy, presented on a fetal template at 32 weeks of gestational age. (**A**) Shows the brain results of first three images. The top three images show the results for the individual cerebellar hubs (from left to right: Hubs 1, 2, 3; at k = 15, p < 0.01), while the lower image shows the combined effect of all three hubs (intersection), revealing a robust effect. (**B**) Shows a scatterplot of the results for the three hubs separately. Created using xjView for SPM 12 in Matlab R2019a. *NASF* Negative Affect and Stress Factor, *FC* Functional connectivity.

In line with our hypothesis, maternal psychological stress/negative affect during pregnancy was associated with variation in fetal cortico-cerebellar connectivity. First, we performed an intersection analyses, combining the association of the three hubs with maternal prenatal NASF. We observed a robust effect of decreased cerebellar to left insula functional connectivity (See Fig. 2). Correlations for separate hubs were computed (Hub1: *r* = − 0.366, *p* = 0.003; Hub2: *r* = − 0.332, *p* = 0.007; Hub3: *r* = − 0.381, *p* = 0.002) and indicated that the association existed for both left (Hub 2 and 3) and right (Hub 1) inferior cerebellum. The effects remained significant after controlling for frame count, movement during scanning, sex of the fetus, gestational age at scanning, maternal age, and maternal income. No significant sex interactions were found for the association between maternal prenatal NASF and cerebellar-insular connectivity (all *p* > 0.05). “Supplemental Materials” include reporting of Family Wise Error (FWE) correction for significant insula effects for each hub.

Following-up analysis of linkage between cerebellar-insular connectivity and individual factors used to derive the omnibus NASF score showed that maternal prenatal perceived stress was most reliably related to cerebellar-insular connectivity (across hubs) and uniquely correlated with hub 1. Correlations between the individual prenatal stress dimensions and fetal cerebellar-insular connectivity are presented in Supplemental Table S2.

### Fetal cerebellar-insular connectivity and toddlers’ sleep problems

Building on the results of decreased left insula to cerebellar fetal functional connectivity in highly stressed mothers, we tested whether cerebellar-insular function connectivity was also associated with child sleep problems. We did not proceed with performing a mediation analyses due to lack of significant associations between fetal cerebellar-insula FC and maternal-reported toddler sleep problems (Hub1: r = − 0.179, p = 0.156; Hub2: r = − 0.075, p = 0.557; Hub3: r = − 0.196, p = 0.121), nor with the actigraphy data. Lack of association suggests there is not likely to be an indirect effect of cerebellar-insular functional connectivity on the association between maternal stress during pregnancy and toddler sleep problems.

### Sensitivity analyses

First of all, sex differences in main predictor and outcome variables were explored by using independent-samples t-tests. We did not find any sex differences for maternal prenatal NASF scores (*t*(62) = 0.221, *p* = 0.826) or mother-reported sleep problems (*t*(62) = − 0.716, *p* = 0.477). Additionally, brain quality measures were not associated with our main variables, maternal prenatal NASF (motion: *r* = − 0.124, *p* = 0.331; frame count: *r* = − 0.032, *p* = 0.799) and child sleep (motion: *r* = 0.188, *p* = 0.138; frame count: *r* = − 0.072, *p* = 0.574). To ensure prematurity is not affecting our results, we reran our analyses excluding all preterm infants with a gestational age ≤ 37 weeks (instead of ≤ 32 weeks). This procedure excluded an additional seven infants (total N = 57). The sex interaction effect for stress and sleep remained significant (B = − 1.320, *t* = − 2.058, *p* = 0.046) and follow-up testing still revealed that the association between maternal NASF during pregnancy and child sleep problems was significant for males only (B = 1.240, *t* = 2.069, *p* = 0.049). The effect for males remained significant after controlling for gestational age at scan, gestational age and weight at birth, maternal age, and maternal postnatal anxiety at age 3 years. Furthermore, correlations between maternal NASF and fetal cortico-cerebellar connectivity in the left insula still yielded similar significant correlations (for all three hubs), while the correlation between child sleep problems and fetal cortico-cerebellar connectivity remained non-significant. Taken together, all sensitivity analyses resulted in similar results, indicating robustness of our findings.

## Discussion

This study presents investigation of the role of fetal cortico-cerebellar functional connectivity (FC) in the association between maternal prenatal negative affect and stress and toddler sleep problems. We found that high maternal negative affect and stress during pregnancy was associated with increased sleep problems in toddlerhood. When exploring sex differences, we found a significant sex interaction indicating that the association was largely driven by males. Additionally, prenatal exposure to maternal negative affect and stress was associated with decreased cerebellar-insula FC in the fetus. The predictive value of fetal cerebellar-insula FC, however, was absent, indicating that fetal cerebellar FC did not mediate the association between maternal negative affect and stress during pregnancy and later child sleep problems. Still, our results present in utero alterations of fetal functional connectivity that are associated with maternal prenatal stress exposure.

Our finding of increased sleep disturbance in toddlers prenatally exposed to maternal stress and negative affect replicates previous reports^13,14^. We found that maternal report of increased sleep problems was replicated in the subsample with direct observation through actigraphy. Our results on mother-reported sleep suggested that males are more at risk of developing sleep problems in response to prenatal exposure to maternal stress and mood later in life. Almost 25% of the variance in male’s mother-reported sleep problems was explained by maternal stress and negative affect during pregnancy. Previous research has reported males as being more sensitive to prenatal influences^46^ and some authors argue that males are more inclined to develop learning difficulties and cognitive concerns after prenatal exposure to maternal stress, while emotional disturbances are more prevalent in prenatally exposed females^45,47^. How these sex-specific differences are related to sleep, is an important question for future research. Moreover, the underlying mechanisms that confer differences between the sexes are largely unknown. Recent research points to several potential pathways, including sex differences in HPA-axis functioning, brain development, and sex-specific adaptation of the placenta^48,49^.

Regarding our objective sleep measurement, we found that children prenatally exposed to higher maternal negative affectivity and stress showed a lower ratio between mean circadian and ultradian power, as measured with actigraphy. Ultradian rhythms—rhythms occurring faster than a day, including sleep cycles—have recently been shown to carry important information about sleep and physiology (for a review, see^50^). Here we found that neither the strength of circadian nor ultradian rhythms alone correlated with early life stress. Our explorative analyses pointed out that children’s variation in circadian power increased when mothers were more stressed during pregnancy. This may have caused the finding of a significant ratio between circadian and ultradian power, in the absence of an association for either one alone. Importantly, it has recently been found that ultradian rhythms by circadian rhythms are indicative of health^51–53^, indicating that the ratio between circadian and ultradian power is of particular interest. Consistent with this observation, we found that higher maternal prenatal stress correlated with a lower ratio between circadian and ultradian power. In this cohort, circadian power and ultradian power correlate, though circadian power rises faster than ultradian power over the population. As a result, this ratio largely reflects a combined power of the two features—higher ultradian power is more likely to occur in individuals with higher circadian power, and the presence of strength in both suggests healthier biological rhythms, which we find is negatively correlated with stress. In other words, children prenatally exposed to higher levels of maternal prenatal stress and mood showed less “healthy” biological rhythms indicative of disturbed sleep cycles. It is important to note that the data were not separated into a training and testing set, as the pool of data was too small for such machine-learning and confirmation approaches. Therefore, while this result matches our expectations about the importance of robust biological rhythms for healthy sleep, further studies are necessary to explore these interactions in a larger population.

In line with previous research (for a review see^20^), our results show altered functional connectivity in the offspring of prenatally stress women. Our results of fetal functional connectivity add important information about timing of this effect by showing that alterations are already observable in utero. Additionally, few studies have explored cortico-cerebellar functional connectivity even though previous researcher have called the cerebellum a “prime target for pediatric neuroimaging”^41^. The cerebellum has a unique protracted developmental trajectories, sexual dimorphism, preferential susceptibility to environmental influences, and its implications in childhood onset disorders such as ADHD and autism, making it a very suitable candidate for studying fetal origins of neurodevelopmental disorders. We found decreased left cerebellar-insula FC in fetuses of mothers experiencing higher negative affectivity and stress during pregnancy. In the adult brain, the insula has positive functional connections with the cerebellum^54^ and altered connectivity of the insula (to other cortical areas) has been related, among other functions, to emotional awareness^55^, saliency^56^, and pain perception^57^. Additionally, altered structural and functional properties of the insula are often reported in maltreated children and adolescents^58–60^. More importantly, thinner insula gray matter volume was found in children prenatally exposed to maternal depression^61^. Future studies should investigate what the implications of decreased fetal functional connectivity of the cerebellum-insula network are for later child behavioral outcomes.

In addition to examining maternal stress and negative affect during pregnancy as one factor score, we also examined the effect of the separate measures of stress. In general, we observed similar pattern of effects, but strength of individual associations varied. Perceived maternal stress during pregnancy and worry showed strongest effects, anxiety and depression showed comparatively lower effects and satisfaction with life showed little or no relationship with child sleep and fetal cerebellar functional connectivity. These findings demonstrate potential loss of detail that can result from collapsing across measures and corroborate prior evidence that varied measures of maternal prenatal stress may differently relate to child (brain) outcomes^62^. Maternal perceived stress and worries may exert different physical and hormonal responses in the pregnant women than depressive symptoms, leading to different effects on the developing fetus—a hypothesis that warrants further investigation in future research.

While we did find an association between fetal cerebellar functional connectivity and maternal stress during pregnancy, there was no indirect effect via cerebellar functional connectivity for the association between maternal prenatal stress and child sleep problems (no mediation). This indicates that the underlying mechanisms of the association are likely not related to the cerebellum-insula connection. One possibility is that other parts of the fetal brain have an important role in mediating sleep, instead of the cerebellum and the insula. Other areas that have been associated with prenatal exposure to maternal stress are the amygdala, hippocampus, and parietal and prefrontal areas^20,21^. Alternatively, maternal prenatal stress may affect child sleep problems via multiple pathways. For example, O'Connor et al.^13^ point to the potential role of glucocorticoids in the association between mother-reported prenatal stress and child sleep problems. Their findings suggest glucocorticoids as a major influence given several reports of a link between the establishment of a diurnal pattern in cortisol and sleeping through the night in infancy. Nevertheless, many researchers have failed to find associations between maternal cortisol and mother-reported stress during pregnancy and/or child outcomes^63,64^. Clearly, more research is necessary to uncover the underlying mechanisms of sleep problems in children prenatally exposed to maternal stress. Such explorations will ideally include alternative brain pathways and also biochemical measures.

Few limitations of the study should be considered. Firstly, the exploration of functional connectivity of the fetal brain in the current study was restricted to the cortico-cerebellar connectivity. We present an initial investigation in to the complex associations between maternal prenatal stress and fetal functional connectome development. Future research should extent to other brain areas, such as the fetal amygdala, hippocampus, and prefrontal cortex. Second, our main analyses was focused on mother-reported sleep problems of their child. Research has shown that current maternal mood can affect ratings of children’s behavioral problems^65^. It is promising that we observed significant associations between maternal prenatal stress and child sleep problems in both subjective and objective data; however, replication of the association between prenatal stress and biological sleep measures in larger samples remains an important aim for future work. Third, it is difficult to disentangle contributions of the postnatal environment to child sleep problems. It is known that prenatal stress may affect maternal parenting^66^, which, in turn, can contribute to child sleep problems. However, the fact that maternal prenatal stress was found to impact functional connectivity of the fetal brain suggests that biological mechanisms are, at least, partly at play. This is corroborated by child sleep behavior measured using actigraphy, as actigraphy captures aspects of physiology, and these data also seemed to implicate biological programming.

In sum, our study has established additional evidence for the notion that child sleep problems may have fetal origins. We found evidence for in utero alterations of fetal functional connectivity in response to prenatal exposure to maternal stress in a sample of predominantly African–American, high-risk mothers and children. More specifically, we found decreased left cerebellar-insula functional connectivity in fetuses of mothers experiencing higher negative affect and stress during pregnancy. Additionally, maternal prenatal negative affect and stress was associated with increased sleep problems in toddlerhood in males. However, we did not observe an indirect effect via altered functional connectivity of the fetal cerebellum to the neocortex for this association. Future research should continue the search for underlying mechanisms and extend the exploration to other brain areas and mediating factors. This information holds the potential to inform innovative interventions to facilitate healthy brain development in utero and stave off deleterious health outcomes related to sleep problems in early childhood, such as increased child emotional and behavior problems and physical health concerns.

## Materials and methods

### Participants

Healthy pregnant women from the Metro Detroit, USA area were recruited in their second or third trimester to take part in a longitudinal fetal magnetic resonance imaging (MRI) brain study between weeks 18 and 36 of pregnancy. Inclusion criteria for participation were: maternal age > 18 years, English as first language, singleton pregnancy, and typical fetal brain development (as assessed by ultrasound and anatomical MRI examination). Some cases completed more than one prenatal MRI exam. For these cases only the later GA scan was included in the current analysis.

After birth, mothers were invited to take part in the longitudinal research protocol, which included a baby research visit, a toddler visit, a preschool visit, and three remote phone assessments. At the time of the current analysis, 118 mothers met eligibility by having completed the toddler follow-up visit. Subsequent exclusions were made for cases with incomplete or missing Child Behavior Checklist (CBCL) data (N = 41) or maternal prenatal stress questionnaires (N = 1), resulting in 76 cases with complete data. Next, cases with extremely low birthweight and/or prematurity (GA < 32 weeks and/or), birthweight < 1500 g; N = 10) were excluded. Finally, two additional children were excluded due to medical complications and/or neurological diagnoses (i.e., cerebral palsy, Autism Spectrum Disorder). A flowchart of our sample selection can be found in the “Supplemental Materials”, see Supplemental Fig. S1.

The final sample consisted of 64 mother–child dyads (39 male). Fetal age at MRI ranged from 20.6 to 39.6 weeks gestational age (GA; range 32.3–39.6, SD = 4.3) and were born at a mean GA of 38.4 (range 34.0–41.7, SD = 1.8). Mother’s age at birth was 24.7 years on average (range 18.3–34.5, SD = 4.4). The majority of the mothers included in the study were African–American, which is representative of the geographic region of the Detroit area. Additionally, our sample is a high-stress, low-resource sample, which is reflected in the depression rates of our participating mothers. Clinical levels of depression (cutoff ≥ 16, for perinatal depression^67^) were reported in 20 out of 64 participating mothers (31.7%). All participating mothers provided written informed consent. Additionally, mothers also provided informed consent for their children to participate in the study. More detailed information of the characteristics of our study sample are included in Table 1.

**Table 1:** Characteristics of mother and child (N = 64).

**Maternal demographics, n (%)**		
Maternal ethnicity		
African–American		53 (82.8)
Asian–American		1 (1.6)
Caucasian		7 (10.9)
Bi-racial		3 (4.7)
Maternal education		
No GED/high-school diploma		24 (37.5)
GED/high-school diploma		14 (21.9)
Some college		22 (34.4)
4-yr college degree		2 (3.1)
2-yr college degree		1 (1.6)
Unreported		0 (0)
Gross annual income		
< $10,000		27 (42.2)
$10,000-$20,000		14 (21.9)
$20,000–$30,000		10 (15.6)
$40,000–$50,000		2 (3.1)
> $50,000		11 (17.2)
Undeclared		4 (6.3)
Marital status		
Single		38 (59.4)
Married/partnered		24 (37.5)
Unreported		1 (1.6)
**Fetal imaging characteristics, range, M (SD)**		
Maternal age at scan (years)	18.2–34.4	24.4 (4.5)
Fetal age at scan (weeks)	20.6–39.6	32.4 (4.4)
Amount of rs-fMRI data analyzed (min)	2.7–11.3	5.4 (1.6)
Translational mean movement (mm)	0.0–0.5	0.3 (0.1)
Rotational mean movement (degrees)	0.0–0.9	0.5 (0.2)
Specific absorption rate (W/kg)		0.2 (0.1)
**Birth outcomes, M (SD)**		
Fetal age at birth (weeks)	34.0–41.7	38.5 (1.8)
Birth weight (g)	1975–4479	3105 (579.6)
Infant gender, male, n (%)		39 (60.9)
**3-year developmental follow-up, M (SD)**		
Child age at assessment (months)	32.5–40.5	35.8 (1.9)

### Study procedure

During the MRI visit, mothers completed self-report questionnaires and reported demographic information. At age 3 years (mean = 35.8 months; SD = 1.3), mothers and their children were invited back to the laboratory for a developmental follow-up visit. During the visit, mothers completed self-report questionnaires, this time also on their children’s behavior. Children participated in the developmental assessment tasks and engaged in interaction tasks with their mother. Additionally, actigraphy measures of sleep were obtained in a subset of study children that reached age 5 years at the time of this analysis (mean = 59.2 months; SD = 4.8). An Actigraph GT3X + Wristband (www.actigraphcorp.com) was worn over the course of contiguous week and weekend days for an average of 6.84 days (SD = 2.10). The wristband was placed by a research assistant during a home visit, worn on the non-dominant arm, and it was explained to the child and the mother that the wristband should not be taken off, except for swimming, bathing, and showering. The study has been approved by the Ethical Review Board of Wayne State University, Detroit, MI, and was conducted in full compliance with the Helsinki Declaration.

### Questionnaires and materials

#### Maternal negative affect and stress factor (NASF)

Maternal stress during pregnancy was measured using five scales that assessed internalizing problems and sources of stress at the time of scanning: Center for Epidemiological Studies Depression Scale (CES-D)^68^, the State Trait Anxiety Inventory (STAI)^69^, the Penn State Worry Questionnaire (PSWQ)^70^, the Perceived Stress Scale (PSST)^71^, and the Satisfaction with Life Scale (SWLS)^72^. Selected instruments were intended to comprehensively assess negative affectivity and stress in participating pregnant women. To reduce the number of variables tested and to extract the overlapping variance of these constructs, we performed explorative factor analysis (EFA) on half of the available sample. Our analyses was performed on all women enrolled in the prenatal MRI scanning protocol (N = 221), including those that did not complete our follow-up protocol. The full sample matches the included sample in terms of social and ethnical background. Moreover, no mean difference was detected between our sample (N = 64) and the rest of the full sample (*t* (217) = 1.643, *p* = 0.102) on the NASF scores.

EFA established that these factors loaded best onto one factor (CFI = 0.98, TLI = 0.96, RMSEA = 0.08, SRMR = 0.03; all factor loadings, p < 0.001). Confirmatory factor analysis (CFA) in the other random half of the sample derived a single factor of maternal negative affect and stress exposure, termed the “Negative Affect and Stress Factor” (NASF). The results indicated that the five scales showed medium to high factor loadings (i.e., correlations) and good fit to a one-factor model (n = 99, CFI = 0.98, TLI = 0.97, RMSEA = 0.06, SRMR = 0.03; factor loadings, p < 0.001). A graphical representation of the factor score, including factor loadings, is presented in Supplemental Fig. S2. For more information on construction of the maternal NASF, see “Supplemental Materials”. The analysis was performed in Mplus vs. 7.2^73^ statistical software. We use the term “NASF” and “maternal prenatal stress factor” interchangeably.

#### Child sleep problems

To measure child sleep problems, mothers completed the 7-item sleep subscale of the Child Behavioral Checklist (CBCL)^74^. The CBCL sleep problems scale is a widely used and well-validated scale in toddlers^75^, which assesses the most commonly reported sleep complaints presented in pediatric sleep clinics and primary care practices. Specifically, the scale includes the following items: resists going to bed at night; does not want to sleep alone; has trouble getting to sleep; wakes up often at night; sleeps less than most children; nightmares; talks or cries out in sleep. Items are rated using a 3-point scale (0 = not true, 1 = somewhat or sometimes true, 2 = very true or often true). The Sleep Problems scale demonstrated adequate internal reliability in the present sample (α = 0.74).

### Functional brain data acquisition and preprocessing

Fetal brain resting-state fMRI data were collected using a Siemens Verio 70-cm open-bore 3-T and a Siemens Flex Abdominal Coil. Images were collected with the following parameters: echo planar imaging (EPI) BOLD, TR/TE 2000/30 ms, axial 4-mm slice thickness, estimated specific absorption rate (SAR) = 0.2 (SD = 0.1); repeated twice. The voxel size was 3.4 × 3.4 × 4 mm^3^. Between 12 and 24 min of fetal resting-state fMRI data were collected per participant.

Resting state fMRI preprocessing followed methods previously described^76–79^. In brief, periods of fetal quiescence were manually identified using FSL image viewer^80^, in which individual segments must consist of at least 20 s (ten frames) of low motion. To ensure that the resulting fetal motion did not contribute to our study outcomes, we checked whether the resulting fetal motion was associated with maternal NASF and/or child sleep outcomes (see below). Brainsuite^81^ was used to manually draw 3D masks for a reference frame from each period of fetal movement quiescence. Masks were then binarized and applied only to frames corresponding to their select segment, and only those data were retained for further analyses. Data quality criteria for fMRI in these analyses required cases have at least 80 volumes of low motion data. For these cases, approximately 50% of data were discarded due to high fetal motion; after motion censoring, an average of 162 frames (M = 5.40, SD = 1.87 min) were retained. As a point of reference, prior fetal fMRI studies have reported censoring 39–56% of total number of acquired fMRI frames^26,78,82,83^.

Subsequent within-segment preprocessing steps included reorientation, realignment and normalization to an average 32 weeks gestational age fetal anatomical template^84^ using Statistical Parametric Mapping (SPM8) software implemented in Matlab (http://icatb.sourceforge.net). To correct for variation in normalization across segments, within-participant normalized images were then concatenated, realigned to the mean BOLD volume, and smoothed using a 4 mm FWHM Gaussian kernel across runs. Further preprocessing was performed in CONN functional connectivity toolbox (v14n)^85^ in MATLAB including linear detrending, nuisance regression using aCompCor of five principal components extracted from a 32-week fetal atlas white matter and CSF mask, six head motion parameters, and band-pass filtering at 0.008–0.09 Hz.

### Seed-based functional connectivity analyses

**Figure 3:**
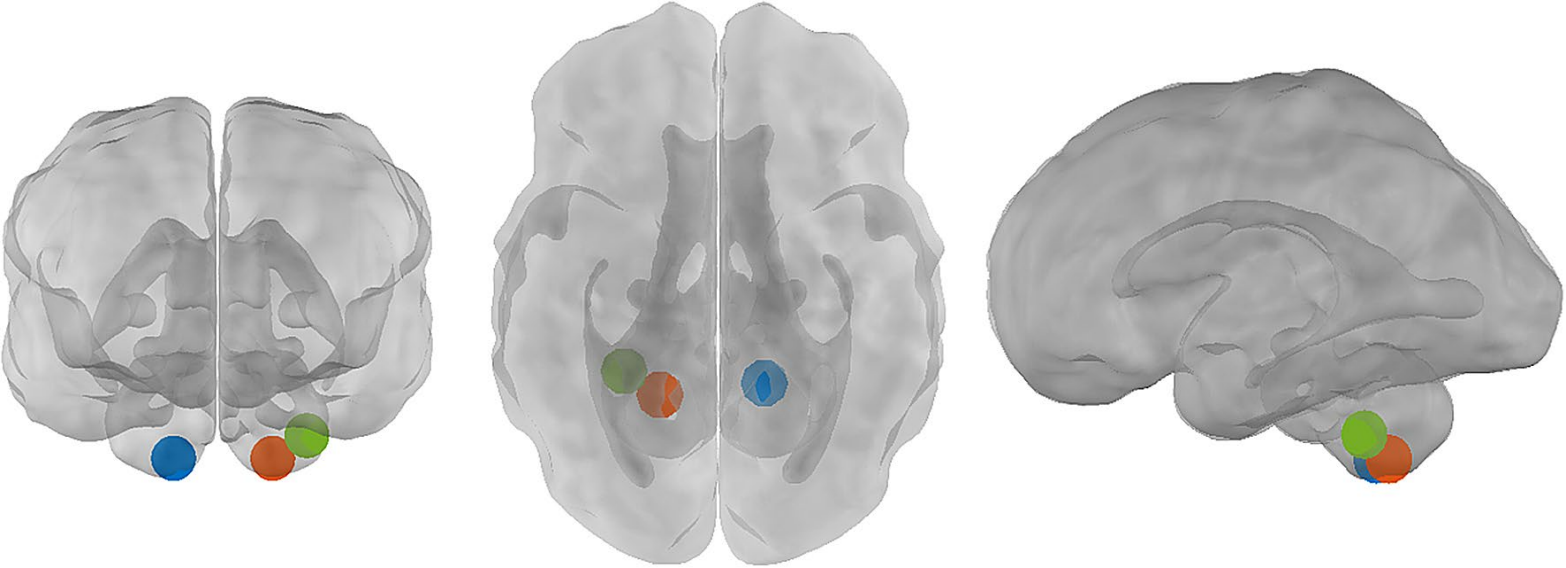
Location of fetal cerebellar hubs. The location of cerebellar hubs is presented on a fetal template at 32 weeks of gestational age. The colours indicate hub 1 (blue), hub 2 (orange), and hub 3 (green). Created using xjView for SPM 12 in Matlab R2019a.

Functional connectivity analyses were carried out with the CONN toolbox for MATLAB (http://www.nitrc.org/projects/conn)^85^. To examine the effect of maternal prenatal stress on fetal functional connectivity of the cerebellum with the cortex, we conducted a seed-to-voxel analysis with the three cerebellar hubs as seed regions. Following prior work, we seeded three hub regions of the fetal functional connectome, identified by graph theoretical analysis^26^. All three hubs are located in the inferior (posterior) part of the cerebellum, which is the largest part of the cerebellum and known to be highly connected with cerebral regions, including parietal and prefrontal cortices^41^. One hub is located in the right hemisphere of the cerebellum, while the other two hubs are located in the left hemisphere. Temporal associations between the mean time courses of all voxels in the seeds with the rest of the brain were estimated using regression analyses. See Table 2 for details on cerebellar hub locations and Fig. 3 for an anatomical representation of the cerebellar hub locations on a fetal template. Note that our previous work on hubs in the human fetal brain network 26 also yielded hubs in other areas of the fetal brain, not located in the cerebellum, such as the inferior temporal gyrus, precentral gyrus, angular gyrus, medial temporal lobe, and the primary visual cortex. Here, however, our focus is on the cerebellar hubs.

**Table 2:** Location of fetal cerebellar hubs.

Hub #	Left/right	Inferior/posterior	Coordinates		
			*x*	*y*	*z*
1	Right	Inferior	8	− 18	− 32
2	Left	Inferior	− 16	− 16	− 28
3	Left	Inferior	− 10	− 20	− 34

### Actigraphy data analyses

Actigraphy data were analyzed in Matlab 2018a using custom processing routines. Data were transformed using wavelets as previously described^51,86,87^. Briefly, wavelets are functions with frequency and location, here, Morse wavelet, that are compared to the data to generate information about frequency composition and stability across the recording window. They are similar to Fourier transforms, but are sensitive to changes in frequency composition across time (non-stationary signals). Daily (circadian) frequencies were isolated as a band between 23 and 25 h periodicity, and ultradian frequencies were isolated as a band between 1 and 3 h periodicities. Features were generated from each individuals’ band characteristics, including: mean circadian power (average strength of the circadian band across the entire window), mean ultradian power (average strength of within-a-day ultradian band across the entire window), and the ratio of the mean circadian/ultradian power. As activity becomes more consolidated to the day time, and decreases in the night time, circadian power increase as a reflection of this dichotomy; in this way circadian power serves as a combined statistical proxy for both sleep consolidation and daily activity. Ultradian characteristics serve as a proxy for biological rhythms associated with hormonal dynamics and sleep cycles, whereas the ratio of the two serves to identify individuals with alterations in one but not both of these measures.

### Statistical approach

To address our research questions, we tested whether maternal NASF was associated with mother-reported child sleep problems at age 3 years by performing a hierarchical regression analysis. The first model included child sleep values as dependent variable and the maternal prenatal stress factor as predictor. We then added an interaction effect of child sex and child sleep problem score (sex*sleep) into the next model and controlled the model for the following confounders in the final model: gestational age at scan, gestational age at birth, birth weight, maternal age, and maternal anxiety at age 3 years postnatal (concurrent maternal anxiety symptoms). Controlling for maternal postnatal anxiety was done to make sure the child sleep problems are not biased by maternal concurrent anxiety symptoms. In addition to our analyses on the mother-reported sleep scores, we explored whether associations existed between maternal NASF and objective measures of child sleep, as measured with actigraphy. To this aim, we performed Pearson’s correlations in the subsample with actigraphy sleep data. Actigraphy measures of interest were mean circadian power (average strength of the circadian band across the entire window), mean ultradian power (average strength of within-a-day ultradian band across the entire window), and the ratio of the mean circadian/ultradian power.

We then investigated the effect of maternal prenatal stress on fetal cortico-cerebellar functional connectivity. Second-level analyses were performed in SPM12 by subjecting the connectivity maps into a regression model with the maternal prenatal NASF scores as the predictor, including gestational age at scan as a covariate. Resultant cerebellar connectivity maps were set to a p < 0.05 (uncorrected) threshold and k-min = 15 and binarized in order to derive a single binary map representing common effects across cerebellar connectivity models (intersection of three sets A ∩ B ∩ C). Intersection of the three models at this threshold yielded a single region of significance in the left insula that became the focus of all subsequent statistical modelling. Functional connectivity values were then extracted from three peaks within the insula conjunction map, for each fetus, in order to test associations with potential confounds, and to assess whether altered fetal functional connectivity mediated the association between maternal stress during pregnancy and child sleep problems. A total of three models were tested in SPSS version 22 (IBM), one for each cerebellar hub. Potential confounds tested in each model included frame count, mean translational and rotational movement, sex of the fetus, gestational age at scanning, maternal age, and maternal income. Models also included an exploratory sex interaction term. While tests of mediation were planned, these were not pursued due to lack of observed significant association between child sleep problems and fetal cerebellar connectivity.

Recent research has shown that different dimensions of maternal distress during pregnancy (anxiety, depression, perceived stress) may have both shared and unique effects on the developing brain in utero^62^. We therefore conducted follow-up analyses for all our tests including the NASF factor, exploring the unique effects of the five individual scales contributing to the omnibus NASF factor score. Results of these follow-up analyses are reported in the “Supplemental Result” section (see “Supplemental Materials”).

Finally, several sensitivity analyses were conducted, to test the robustness of our findings. First of all, we tested whether there were any sex differences in our main predictors with two sampled t-tests. Next, we checked whether motion differences were confounding observed effects. Fetal movement effects are kept to a minimum by only including frames of low motion. However, some motion may remain. In order to check confounding motion effects, we computed Pearson’s correlations between brain quality measures (average XYZ mean and the frame count) and our outcome variables (prenatal NASF and child sleep problems).

## Supplementary Information

Supplementary Information.
