# Supplementary material for: Maternal stress during pregnancy alters fetal cortico-cerebellar connectivity in utero and increases child sleep problems after birth
Source: Sci Rep. 2021 Jan 26;11:2228. doi: 10.1038/s41598-021-81681-y (PMC7838320; doi:10.1038/s41598-021-81681-y)
Supplement: Supplementary file 1 — Supplementary Information. [file 41598_2021_81681_MOESM1_ESM.pdf]

Maternal stress during pregnancy alters fetal cortico-cerebellar connectivity in utero and increases child sleep problems after birth

Marion I. van den Heuvel[1*], Jasmine L. Hect[2], Benjamin L. Smarr[3], Tamara Qawasmeh[2], Lance J. Kriegsfeld[3,4], Jeanne Barcelona[5], Kowsar E. Hijazi[2], Moriah E. Thomason[2,6,7]

SUPPLEMENTAL RESULTS

Whole brain effects of NASF related variation in cerebellar connectivity

Multiple comparisons were addressed at the individual SPM model level using full brain cluster level correction and small volume correction for the anterior insula. After applying whole brain Family Wise Error (FWE) correction, one significant cluster (k=176) at -8, 14, -10 was observed for hub 1, t = -3.72, p-corrected (p-corr)= 0.04, which extended into the left insula area of interest. FWE within the insula volume (based on anatomical boundaries) was significant for hubs 1 (t = -3.67, p-corr = 0.041) and 3 (t = -3.65, p-corr = 0.044), and showed a trend for hub 2 (t = -2.87, p-corr = 0.060). These results confirm that NASF related differences in cerebellar-insula connectivity were observed across models, prior to derivation of the binary intersection map used in subsequent analyses.

Prenatal exposure to maternal prenatal stress is related to child sleep problems

Follow-up correlations with individual stress/negative affect scales indicated that the strongest association was found for maternal perceived stress during pregnancy (PSST: r=0.339, p=0.007), while no association was found for maternal prenatal depression and child sleep (r=0.219, p=0.089). For the Actigraphy sleep measures, we found significant correlations for maternal worry during pregnancy (r=-0.402, p=0.046) and maternal depression (r=-0.418, p=0.047) with the ratio of circadian and ultradian power.

SUPPLEMENTAL METHODS

Construction of the maternal negative affect and stress factor (NASF)

Based on the results of the exploratory factor analysis, we specified a single factor, termed “maternal negative affectivity and stress factor (NASF)”. All five scales showed medium to high factor loadings (0.32 – 0.76) and good fit to a one-factor model, χ^2=19.11 (df=9), p=0.02; CFI

=0.97; TLI=0.96; RMSEA=0.08; SRMR=0.03, with each scale loading significantly on the factor ($p < 0.001$). See Figure S1 for all factor loadings. The analysis was performed in Mplus vs. 7.2 statistical software.

After establishing the one-factor score, we examined whether the derived single factor was associated with other demographic variables in the sample, which also had potential to influence maternal stress during pregnancy and child sleep issues. We tested this in a regression model that examined whether demographic factors (i.e., age, education level, and income) were uniquely related to prenatal stress exposure. Additionally, we tested whether the maternal prenatal stress factor was associated with criterion health variables (i.e., maternal diet, exercise and sleep) with Pearson's correlations. We observed that higher scores on the cumulative prenatal stress latent factor showed expected significant correlations with criterion health variables (diet: r=-0.303, p=0.019; exercise: r=-0.277, p=0.035, sleep: r=-0.294, p=0.025), but were not associated with maternal education level (B=-0.089, t=-0.773, p=0.443), income (B=0.16, t=0.318, p=0.752), or age (B=0.001, t=0.047, p=0.963) in our high-risk sample. Thus, the factor we derived was not a reflection of simply being low-resource, but rather, provided a valid index of the significant individual differences in psychological health and well-being, and experience of stress.

SUPPLEMENTAL TABLES

Table S1. Correlations between maternal prenatal stress dimensions and sleep measures

Prenatal stress dimension	CBCL Sleep problems	Actigraphy – mean circadian power	Actigraphy – mean ultradian power	Actigraphy – ratio circadian/ultradian
Perceived stress	**0.279***	0.041	0.053	-0.338^
Life satisfaction	-0.098	-0.056	-0.066	0.315
Worry	**0.339****	-0.127	-0.125	**-0.402***
Anxiety	0.113	0.069	-0.097	-0.251^
Depression	0.219	0.030	0.002	**-0.418***

Note. *^p < 0.10; *p < 0.05; **p < 0.01; CBCL = Child behavioral Checklist*

Table S2. Correlations between maternal prenatal stress dimensions and fetal insular-cerebellar connectivity

Prenatal stress dimension	Hub 1	Hub 2	Hub 3
Perceived stress	**-0.339****	**-0.263***	**-0.285***
Life satisfaction	0.184	**0.287***	**0.318***
Worry	-0.241^	**-0.256***	**-0.304***
Anxiety	-0.238^	**-0.375****	**-0.389****
Depression	-0.221^	-0.237^	-0.244^

Note. *^p < 0.10; *p < 0.05; **p < 0.01*

SUPPLEMENTAL FIGURES

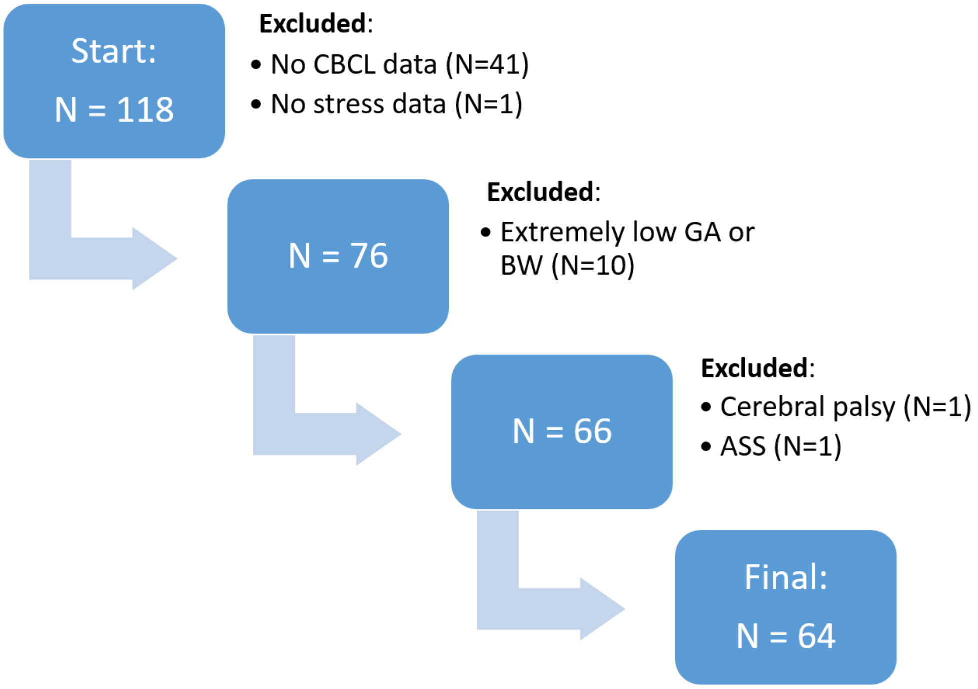

Supplemental Figure S1. Flowchart of sample selection (N=64). Note: CBCL = Child Behavioral Checklist; GA = Gestational age at birth; BW = Birth Weight;

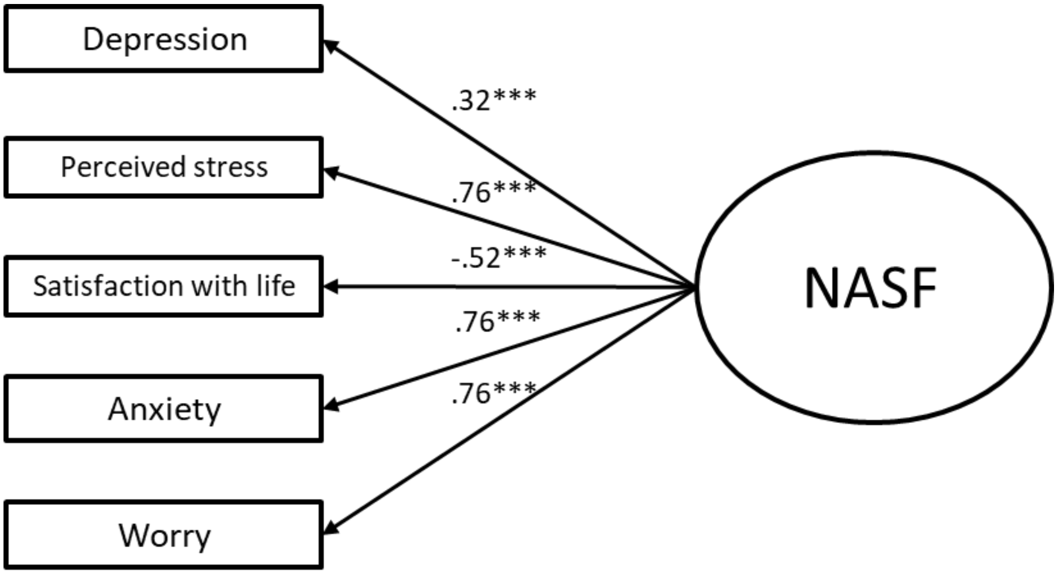

Supplemental Figure S2. Graphical representation of NASF including factor loadings. All five scales show high loadings and good fit to a one-factor model (n = 99, CFI = .98, TLI = .97, RMSEA = .06, SRMR = .03; factor loadings, $p < .001$). *Note*: NASF = Negative Affect and Stress Factor.
